# Suboptimal protein intake for hypocaloric diet needs while using glucagon-like peptide-1 receptor agonists

**DOI:** 10.1080/15502783.2025.2550139

**Published:** 2025-08-25

**Authors:** Brittany Johnson, Tyler McGlasson, Olena Thomas, Rachel Kreider, Rachel Jones

**Affiliations:** GNC Holdings, LLC, Research & Development Department, Pittsburgh, PA, USA

**Keywords:** Diet, GLP-1, weight loss, fat loss, body composition

## Abstract

**Background:**

The primary aim of this study was to evaluate protein intake of individuals while using a Glucagon-like Peptide-1 Receptor Agonist medication (GLP-1RA). Recommended protein intake during hypocaloric diets is 1.2–2.0 g/kg/d based on adjusted body weight to help preserve muscle mass.

**Methods:**

Sixty adults, males (*n* = 13) and females (*n* = 47), using a GLP-1RA for at least one month for weight loss participated in a cross-sectional study (mean age = 49 ± 13; body mass: males = 112, females = 96.9 kg; adjusted body mass: males = 84, females = 65 kg; height: males = 176.8, females = 164 cm). A 3-day food record was collected to determine average protein intake compared to individual protein needs (1.2–2.0 g/kg/d) based on adjusted body weight (greater than 115% ideal body weight), calculated from self-reported height and current body weight. Self-reported changes in exercise habits (less often, no change, or more often) since beginning GLP-1RA were also assessed.

**Results:**

Average intake was 1,763 ± 669 kcal/d and 17.5% of total calories were from protein. Within this sample, 26 (43%) participants consumed at least 1.2 g/kg/d of protein, 6 (10%) participants consumed at least 1.6 g/kg/d, and 3 (5%) participants consumed at least 2.0 g/kg/d. A paired sample t-test revealed statistically significant differences between average 3-day protein intake (males = 88.1 ± 30.1; females = 74.4 ± 28.6 g/d) and calculated protein needs of 1.6 g/kg/d (males = 134.8 ± 17.5; females = 103.8 ± 18.8 g/d) and 2.0 g/kg/d (males = 168.5 ± 21.9; females = 129.7 ± 23.5 g/d), *p* < 0.001, but not significant for 1.2 g/kg/d (males = 101.1 ± 13.1; females = 77.8 ± 74.4 g/d) (*p* = 0.131). Only 53% of the population reported exercising more often than before beginning the GLP-1RA. A one-way ANOVA showed no significant difference in changed exercise habits and calorie (*p* = 0.228) or protein intake (*p* = 0.245) from the 3-day food records.

**Conclusions:**

The analysis found suboptimal protein intake for hypocaloric diets in this population using a GLP-1RA. Given the high risk of sarcopenic obesity and metabolic adaptations after significant weight loss, future clinical trials are needed to assess optimal dietary patterns, protein dosing (g/kg/d) and timing, protein supplementation, and resistance training interventions to better promote muscle health outcomes during weight loss.

